# Irradiance, Photofrin^®^ Dose and Initial Tumor Volume are Key Predictors of Response to Interstitial Photodynamic Therapy of Locally Advanced Cancers in Translational Models

**DOI:** 10.1111/php.13207

**Published:** 2020-02-18

**Authors:** Emily Oakley, David Bellnier, Alan Hutson, Hannah Cooper, Michael Habitzruther, Sandra Sexton, Leslie Curtin, Lawrence Tworek, Matthew Mallory, Barbara Henderson, Gal Shafirstein

**Affiliations:** ^1^ Photodynamic Therapy Center Roswell Park Comprehensive Cancer Center (Roswell Park) Buffalo NY; ^2^ Department of Cell Stress Biology Roswell Park Buffalo NY; ^3^ Department of Biostatistics and Bioinformatics Roswell Park Buffalo NY; ^4^ Laboratory Animals Shared Resources Roswell Park Buffalo NY

## Abstract

The objective of the present study was to develop a predictive model for Photofrin^®^‐mediated interstitial photodynamic therapy (I‐PDT) of locally advanced tumors. Our finite element method was used to simulate 630‐nm intratumoral irradiance and fluence for C3H mice and New Zealand White rabbits bearing large squamous cell carcinomas. Animals were treated with light only or I‐PDT using the same light settings. I‐PDT was administered with Photofrin^®^ at 5.0 or 6.6 mg kg^−1^, 24 h drug‐light interval. The simulated threshold fluence was fixed at 45 J cm^−2^ while the simulated threshold irradiance varied, intratumorally. No cures were obtained in the mice treated with a threshold irradiance of 5.4 mW cm^−2^. However, 20–90% of the mice were cured when the threshold irradiances were ≥8.6 mW cm^−2^. In the rabbits treated with I‐PDT, 13 of the 14 VX2 tumors showed either local control or were cured when threshold irradiances were ≥15.3 mW cm^−2^ and fluence was 45 J cm^−2^. No tumor growth delay was observed in VX2 treated with light only (*n* = 3). In the mouse studies, there was a high probability (92.7%) of predicting cure when the initial tumor volume was below the median (493.9 mm^3^) and I‐PDT was administered with a threshold intratumoral irradiance ≥8.6 mW cm^−2^.

## Graphical abstract

In this study, we applied our image‐based finite element method for simulating intratumoral irradiance and fluence to guide the light delivery and dosimetry during Photofrin®‐mediated I‐PDT of locally advanced squamous cell carcinomas in C3H mice and New Zealand White rabbits. Our results demonstrated that for the same threshold intratumoral fluence, the intratumoral threshold irradiance along with the initial tumor volume is a key parameter in predicting tumor response to Photofrin®‐mediated I‐PDT.

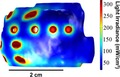

## Introduction

Interstitial photodynamic therapy (I‐PDT) is a promising alternative treatment for patients with deep‐seated or locally advanced cancers (≥10 mm in thickness) that either failed to respond or were not amenable for standard of care therapies (surgery, radiation therapy and chemotherapy) 1, 2. In I‐PDT, the tissue‐localized photosensitizing drug (i.e. photosensitizer, PS) is activated with laser light delivered through multiple diffusing optical fibers which can be inserted directly into the target tumor volume through sterilized transparent catheters 3.

Several clinical studies have applied I‐PDT as a palliative treatment option for patients with locally advanced cancers of the head and neck and prostate 4, 5, 6, 7, 8, 9, 10. From these studies, interpatient variability in response demonstrated the need for individualized treatment planning and proper dosimetry for I‐PDT. Several investigators have reported on the use of the intratumoral fluence (J cm^−2^) to plan and predict tumor control during I‐PDT of prostate cancer 9, 10, 11, 12. Recently, we demonstrated through the use of our finite element method (FEM) for simulating light propagation that in addition to the fluence, the irradiance (mW cm^−2^) is a key parameter to achieving tumor control and cures from I‐PDT with Photofrin^®^ 3. In that study, we reported 70–90% cures from Photofrin^®^‐mediated I‐PDT in C3H mice with locally advanced squamous cell carcinoma VII (SCCVII) when the FEM‐simulated irradiance and fluence were, respectively, ≥8.4 mW cm^−2^ and ≥45 J cm^−2^. We also observed local tumor control and cures from Photofrin^®^‐mediated I‐PDT in 4 of the 5 New Zealand White (NZW) rabbits with VX2 carcinoma when the FEM‐simulated irradiance and fluence were, respectively, ≥16.5 mW cm^−2^ and ≥45 J cm^−2^.

Here, we present new preclinical data from experimental animals (C3H mice and NZW rabbits) treated with consecutive light illumination in which the total therapeutic light dose is delivered in 2–3 treatment sessions with no more than 5 min between each session. By applying consecutive light illumination in the treatment of large tumors, we can carefully monitor and adjust the light administered during treatment to deliver a prescribed fluence and irradiance. This is achieved by inserting multiple catheters into the target tumor volume and margins and by changing the location of the treatment fibers and light dosimetry fibers for each treatment session.

In this study, we used our FEM to simulate the fluence and irradiance distribution throughout the large SCCVII and VX2 tumors as a result of consecutive light illumination. SCCVII tumors were treated with Photofrin^®^‐mediated I‐PDT at subtherapeutic and therapeutic irradiances. Our results from these mouse studies were combined to develop a predictive model for Photofrin^®^‐mediated I‐PDT based on the combination of irradiance, Photofrin^®^ dose and initial tumor volume. We used our FEM to develop individual light regimens for each of the large VX2 tumors treated with Photofrin^®^‐mediated I‐PDT. In the rabbit study, our light dosimetry system was used to confirm that the prescribed threshold fluence and irradiance were delivered within the tumor volume and margins.

## Materials and methods

All animal procedures were conducted in accordance with a protocol approved by the Institutional Animal Care and Use Committee at Roswell Park Comprehensive Cancer Center.

### Computer simulations of light propagation in tissue

A detailed description of our previously developed and validated FEM for simulating light propagation in tissue during I‐PDT is given in Oakley et al. 2015, 2017 13, 14. Briefly, we used a FEM to solve to the three‐dimensional (3‐D) time‐dependent diffusion approximation (Eq. (1)) as derived from the equation for radiative transfer:

(1) $$\frac{1}{c_{n}}\left(\frac{\partial }{\partial t}\Phi \left(\text{x, y, z, t}\right)-\nabla \left(\alpha_{n}\nabla \Phi \left(\text{x, y, z,t}\right)\right)\right)=-\mu_{a}\Phi \left(\text{x, y, z, t}\right)$$

Where:

(2) $$\alpha_{n}=c_{n}\cdot {\left[3\left(\mu_{a}+\left(1-g\right)\mu_{s}\right)\right]}^{-1}$$

Φ(x, y, z, t) is the photon flux (P_n_ m^−2^ s^−1^), where P_n_ is the number of photons, α_n_ is the optical diffusion coefficient (m^2^ s^−1^) of tissue n, μ_a_ and μ_s_ are the linear absorption and scattering coefficients (1/m) of tissue, g is the anisotropy factor, and c_n_ is the speed of light in tissue n.

In I‐PDT, the laser light is provided via cylindrical light‐diffusing fibers (between 1 and 5 cm diffuser length) placed within transparent plastic catheters that are percutaneously inserted into the tumor volume. In our FEM, we model laser light delivered from the diffuser fibers as a flux of diffused photons emitted from the outside surface of the catheter. This boundary condition is given as follows:

(3) $$\frac{P_{\text{laser}}c_{o}}{\left(h_{p}v_{l}\right)}=-\alpha_{n}\nabla \Phi \left(\text{x, y, z, t}\right)$$

P_laser_ is the input light irradiance (W m^−2^) per source diffuser fiber, c_o_ is the speed of light in a vacuum, 3 × 10^8^ m s^−1^, and h_p_ is Planck’s constant (6.6260957 × 10^−34^ J s). v_l_ is the laser light frequency (1/s) defined as c_o_ divided by λ (the wavelength of the laser light).

In our FEM approach, an image visualization and analysis software package (Simpleware, Exeter, UK) was used to create 3D computer models representative of the tumors with surrounding anatomy (noncancerous tissue, bone, arteries, etc.) from either magnetic resonance imaging (MRI) or computed tomography (CT) scans. These 3D models were exported to a finite element analysis software, Comsol 5.2a (Comsol Inc., Burlington, MA). In Comsol, 3D cylindrical representations of the catheters containing the light‐diffusing fibers were virtually placed into the tumor geometry. The number and location of the catheters were selected based on the size and location of the tumor. The resulting light distribution throughout the entire tumor geometry was computed by solving Eq. (1) for the photon flux using the appropriate initial and boundary conditions. The optical properties used for the FEM simulations are given in Table 1. These optical properties were measured from a representative SCCVII tumor and VX2 tumor at λ = 630 nm (the wavelength of light used to activate Photofrin^®^) using a commercial real time optical reflectance spectroscopy device (Zenascope™ PC1; Zenalux Biomedical, Durham, NC). Our FEM solution was then applied to compute the intratumoral irradiance and fluence for different input intensities (mW cm^−1^) and energies (J cm^−1^) per fiber. In our FEM simulations, we assumed either simultaneous light illumination or consecutive light illumination. In simultaneous light illumination, the therapeutic light dose was delivered from all the catheters containing light diffuser fibers at the same time therefore only one FEM simulation was needed. In consecutive light illumination, the total therapeutic light dose was delivered in 2–3 treatment sessions, with no more than 5 min between each session. In each session, light was delivered from either 1 catheter (if only 2 catheters were planned for light delivery) or from 2–7 catheters (if more than 8 catheters were planned for light delivery). In each session, the light diffuser fibers were moved among the catheters. A FEM simulation was performed for each treatment session. In each session, the irradiance and fluence at each voxel within the tumor volume was computed. These data were used to reconstruct a solution file that included the maxima irradiances and the total fluence at each voxel over the entire treatment time. Then, a search algorithm in Matlab^®^ (Matlab^®^ 2012a; MathWorks Inc., Natick, MA) was used to find the minimum irradiance and fluence throughout the tumor volume. In the remainder of the text, the minimum irradiance and the minimum fluence that 100% of the target tumor volume receives will be referred to as, respectively, the threshold irradiance and threshold fluence.

**Table 1:** Properties used for FEM computation of light propagation.

	Input Data	Description
SCCVII Tumor	*μ*_a_ = 1.45 (1/cm)	Tissue linear absorption coefficient
	*μ*_s_ = 38 (1/cm)	Tissue linear scattering coefficient
	g = 0.8	Optical anisotropy factor
VX2 Tumor	*μ*_a_ = 0.093 (1/cm)	Tissue linear absorption coefficient
	*μ*_s_ = 25.8 (1/cm)	Tissue linear scattering coefficient
	g = 0.639	Optical anisotropy factor

### Consecutive light illumination interstitial photodynamic therapy of locally advanced SCCVII

A detailed description of the murine tumor model used in this study was previously described in Shafirstein et al. 2018 3. Briefly, female C3H mice were inoculated with SCCVII squamous cell carcinoma (10^6^ cells), which is a common murine tumor model used for studying head and neck cancer 15, 16. When tumors reached a volume of about 400–700 mm^3^ thereby mimicking locally advanced tumors, the mice were treated with either no drug (light only) or with Photofrin^®^ administered in a single bolus, via intravenous tail vein injection 24 h prior to light delivery at a concentration of either 5.0 or 6.6 mg kg^−1^. Mice were treated while under isoflurane (3–5%) with oxygen anesthesia. The laser light was delivered through two 0.98 mm diameter optical fibers with a 2‐cm cylindrical diffuser (RD20; Medlight SA, Ecublens, Switzerland) connected to a 1.0 W diode‐lasers that emit 630 ± 3 nm light (ML6500‐630; Modulight Inc., Tampere, Finland). The RD20 fibers were placed in sterilized transparent catheters (18 G shielded IV catheter; Becton, Dickinson and Company, Franklin Lakes, NJ) that were inserted through the tumor volume along its longest axis 6 ± 1 mm apart. A MRI of a representative SCCVII tumor post‐catheter insertion was used for our FEM simulations of light irradiance and fluence. The 630‐nm laser light was administered consecutively with an intensity of either 100, 160 or 200 mW cm^−1^ per fiber. At the beginning of the treatment the tumor was illuminated with one RD20 fiber. Once the total dose of 1080 J (540 J cm^−1^ from the 2 cm cylindrical diffuser fiber) was administered, the laser light was turned off for no more than 5 min, and then, the tumor was illuminated with only the second RD20 fiber to administer an additional 1080 J (540 J cm^−1^ from the 2 cm cylindrical diffuser fiber). Tumor response was followed for up to 60 days, and a cure was defined as complete regression following the I‐PDT and no evidence of tumor at least 60 days post‐treatment, as previously reported (Shafirstein et al. 2018). Animals were euthanized either at 60 days post‐treatment or when the tumor volume reached ≥2000 mm^3^.

### Interstitial photodynamic therapy of locally advanced VX2 carcinoma

A detailed description of the rabbit model used in this study is given in Shafirstein et al. 3. Briefly, VX2 carcinoma was surgically implanted in either the sternomastoid muscle of the neck or the biceps femoris of the thigh of NZW rabbits. Tumors were treated at a volume of approximately 3000–15 000 mm^3^. Noncontrast‐enhanced CT (LightSpeed VCT; GE Healthcare) scans of the rabbit with surface fiducial CT markers (IZI Medical Products, Owings Mills, Maryland) were taken 1–2 days prior to treatment. These images were used to develop individualized FEM‐based treatment plans indicating the number and location of source diffuser fibers needed to deliver a threshold of 8.4 mW cm^−2^ and 45 J cm^−2^ to 100% of the tumor volume. In 14 rabbits (*n* = 6 with VX2 implanted in the neck and *n* = 8 with VX2 implanted the thigh), a single bolus of 5 mg kg^−1^ Photofrin^®^ was administered intravenously via a catheter in the auricular vein 24 h prior to treatment. The other five rabbits (*n* = 3 with VX2 implanted in the neck and *n* = 2 implanted in the thigh) were treated with light only (no photosensitizer). The rabbits were treated under general anesthesia with isoflurane (2% in oxygen) inhalation. The CT fiducial markers were used to guide the placement of the sterilized transparent catheters (15 G shielded IV catheter; Becton, Dickinson and Company, Franklin Lakes, NJ) according to the treatment plan, as detailed in Oakley et al. 2017 14. The laser light was delivered through 0.98 mm diameter optical fibers with either a 1‐, 2‐, 2.5‐, 3‐, 4‐ or 5‐cm cylindrical diffuser (Medlight SA, Ecublens, Switzerland) each connected to a 1.0 W diode‐laser that emit 630 ± 3 nm light (ML6500‐630, Modulight Inc., Tampere, Finland). These laser source diffuser fibers were placed in the catheters based on the treatment plan. Additional catheters were inserted in order to monitor the light dose at the margins of the tumor. Isotropic detection fibers (IP85; Medlight SA, Ecublens, Switzerland) were placed in at least 3–5 of the vacant catheters. The IP85 detection fibers were connected to USB spectrometers and our light dosimetry system (described in Shafirstein et al. 2016 17) in order to monitor the delivered irradiance and fluence during treatment. The light dosimetry was calibrated in air, and a correction factor was applied that takes into account the difference in the index of refraction between air and tissue following the method described in Marijnissen and Star 1996, as previously reported in Oakley et al. 2015. The treatment time was based on these dosimetry measurements. For 17 out of the 19 rabbits, the total treatment was divided into sessions of consecutive light illumination.

### Statistical analysis

A total sample size of *n* = 94 mice were utilized in the statistical analysis. Two primary endpoints were examined: time‐to‐tumor volume reaching ≥2000 mm^3^ and cure rate. The time‐to‐event data were analyzed using a Cox proportional hazards accounting for interval censored data 18. Corresponding hazard ratios (HRs) and 95% confidence intervals (CIs) were calculated. Survival curves were estimated using interval‐censored nonparametric maximum‐likelihood approaches 19. Cure rate was modeled using logistic regression. Corresponding odds ratios (ORs) and 95% confidence intervals were calculated. Covariates examined in each model were tumor volume dichotomized at the median, baseline tumor volume and input light intensity. Test of covariates was two‐sided and tested at level alpha = 0.05. The receiver operating characteristic curve (ROC) and the area under the ROC curve (AUC) with the 95% confidence interval were derived from the logistic regression modeling. All analyses were carried out using SAS 15.1 (SAS Institute Inc., Cary, NC, US). Analyses of the rabbit data are primarily descriptive in nature.

## Results

### Consecutive light illumination interstitial photodynamic therapy of locally advanced SCCVII

**Figure 1:**
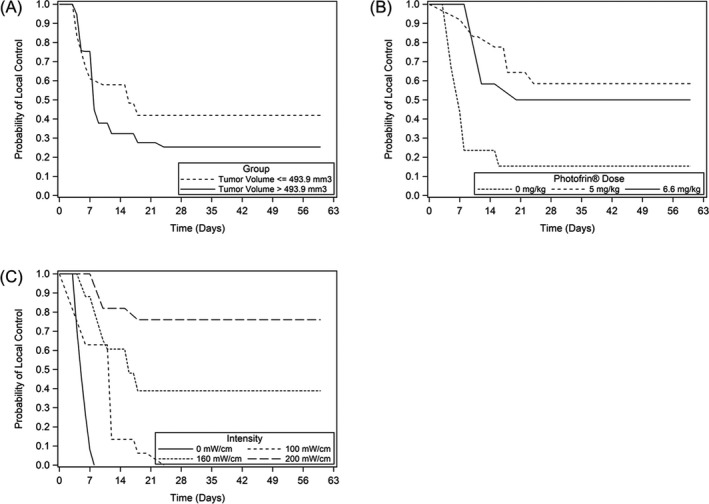
Survival curves for C3H mice treated with consecutive light illumination. (A) shows the survival as a function of time and initial tumor volume. (B) shows the survival as a function of time and Photofrin^®^ dose. (C) shows the survival as a function of time and intensity per fiber.

**Figure 2:**
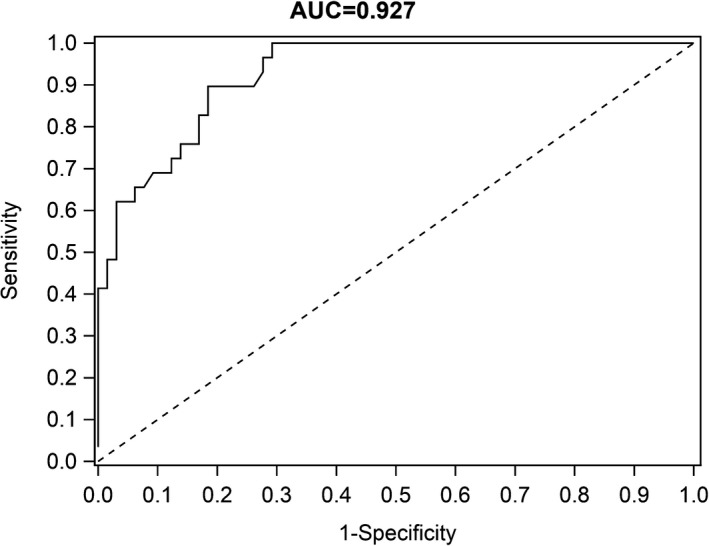
The receiving operating characteristic curve (ROC) and the area under the ROC curve (AUC) derived from logistic regression modeling. This analysis assesses the accuracy of predicting a cure *versus* no cure based on Photofrin^®^ dose, irradiance and initial tumor volume being below or above the median tumor volume treated in this study.

In this study, the impact of the irradiance on treatment response to Photofrin^®^‐mediated I‐PDT was further investigated by treating C3H mice with locally advanced SCCVII tumors using two 2 cm light diffuser fibers each delivering consecutively 540 J cm^−1^ per fiber and either 100, 160 or 200 mW cm^−1^ per fiber. The tumor volume measured just before treatment (initial tumor volume) was in the range of 369–730.3 mm^3^, with a mean volume of 493.9 mm^3^. Table 2 provides a summary of the results obtained from our FEM simulations and *in vivo* studies. Time for the tumor volume to reach ≥2000 mm^3^ was a function of Photofrin^®^ dose (*P* = 0.006: HR = 0.835, 95% CI (0.734, 0.949)) and irradiance (*P* < 0.001: HR = 0.984, 95% CI (0.978, 0.989)) with being above the median baseline tumor volume (>493.9 mm^3^) not significant, but suggestive (*P* = 0.06: HR = 1.74, 95% CI (0.970, 0.3.125)). This indicates that increased Photofrin^®^ dose and irradiance delayed the time for the tumor volume to reach ≥2000 mm^3^ as illustrated in Fig. 1A–C. No cures were obtained in mice treated with consecutive light illumination and 100 mW cm^−1^, 540 J cm^−1^ per fiber when the computed threshold irradiance was 5.4 mW cm^−2^. When the threshold irradiance was increased to 8.6 and 10.8 mW cm^−2^ by increasing the input intensity per fiber to, respectively, 160 and 200 mW cm^−1^, between 20% and 90%, cure rate was obtained. It is important to note that at 160 and 200 mW cm^−1^, cures were obtained in both mice treated with Photofrin^®^ and mice treated with light only. We previously reported that at our effective light regimen for Photofrin^®^‐mediated I‐PDT with simultaneous light illumination, light‐induced tissue heating to temperatures ≥60°C (thermal ablation) occurred 3. In that study, this thermal ablation alone, although less effective than Photofrin^®^‐mediated I‐PDT, resulted in some cures (up to 40%) when treating locally advanced SCCVII. In the current study, we ran a statistical analysis comparing tumor response in mice treated with Photofrin^®^‐mediated I‐PDT (5 mg kg^−1^ or 6.6 mg kg^−1^ Photofrin^®^) to those treated with light only. Fig. 1B shows the probability of obtaining local tumor control as a function of the time post‐treatment and Photofrin^®^ dose. From the figure, we can see an increase in the probability of local control (up to 60%) when Photofrin^®^ is administered to the animal. In addition to the Photofrin^®^ dose, we also evaluated the probability of obtaining local control as a function of initial tumor volume and irradiance (see Fig. 1A and C, respectively). In Fig. 1A, we observe that there is a higher probability (40% *vs* 25%) of obtaining local tumor control when treating tumors smaller than the median. In Fig. 1C, we observe that as the irradiance is increased, we also increase the probability of local tumor control (up to 76%). Next, we aimed to develop a predictive model for Photofrin^®^‐mediated I‐PDT based on the initial tumor volume, Photofrin^®^ dose and irradiance. Because the input energy per fiber and FEM‐simulated minimum fluence were constant throughout the study, these parameters were not included in our analysis. The statistical analysis revealed that cure rate was a function of Photofrin^®^ dose (*P* = 0.037: OR = 1.316, 95% CI (1.016, 1.703)) and irradiance (*P* < 0.001: OR = 1.066, 95% CI (1.029, 1.104)) and being above the median tumor volume (>493.9 mm^3^) (*P* = 0.0015: OR = 0.986, 95% CI (0.975, 0.997)). These results indicate that increased baseline tumor volume leads to a lower cure rate, while increases in Photofrin^®^ dose and irradiance lead to increased cure rates. In terms of predicting a cure as a function of these three factors, we generated an ROC curve shown in Fig. 2. The AUC for the ROC curve was 0.927, and the 95% CI was (0.88, 0.98) indicating that the model accurately predicted 92.7% of the cures and noncures across all mice. In terms of a decision cut point, if we use the point closest to the upper left corner of the ROC curve we arrive at an estimated sensitivity = 0.86 and 1‐specificity = 0.18.

**Table 2:** Summary of results for I‐PDT of locally advanced SCCVII tumors.

Input Intensity (mW cm^−1^)	Input Energy (J cm^−1^)	FEM‐Simulated Threshold Irradiance (mW cm^−2^)	FEM‐Simulated Threshold Fluence (J cm^−2^)	Photofrin^®^ Dose (mg kg^−1^)	# of Mice	Cure Rate
100	540	5.4	45.5	0	11	0%
				5	5	0%
160	540	8.6	46	0	10	20%
				5	10	30%
				6.6	12	41.7%
200	540	10.8	45.5	0	10	60%
				5	15	86.7%

### Interstitial photodynamic therapy of locally advanced VX2 carcinoma

**Figure 3:**
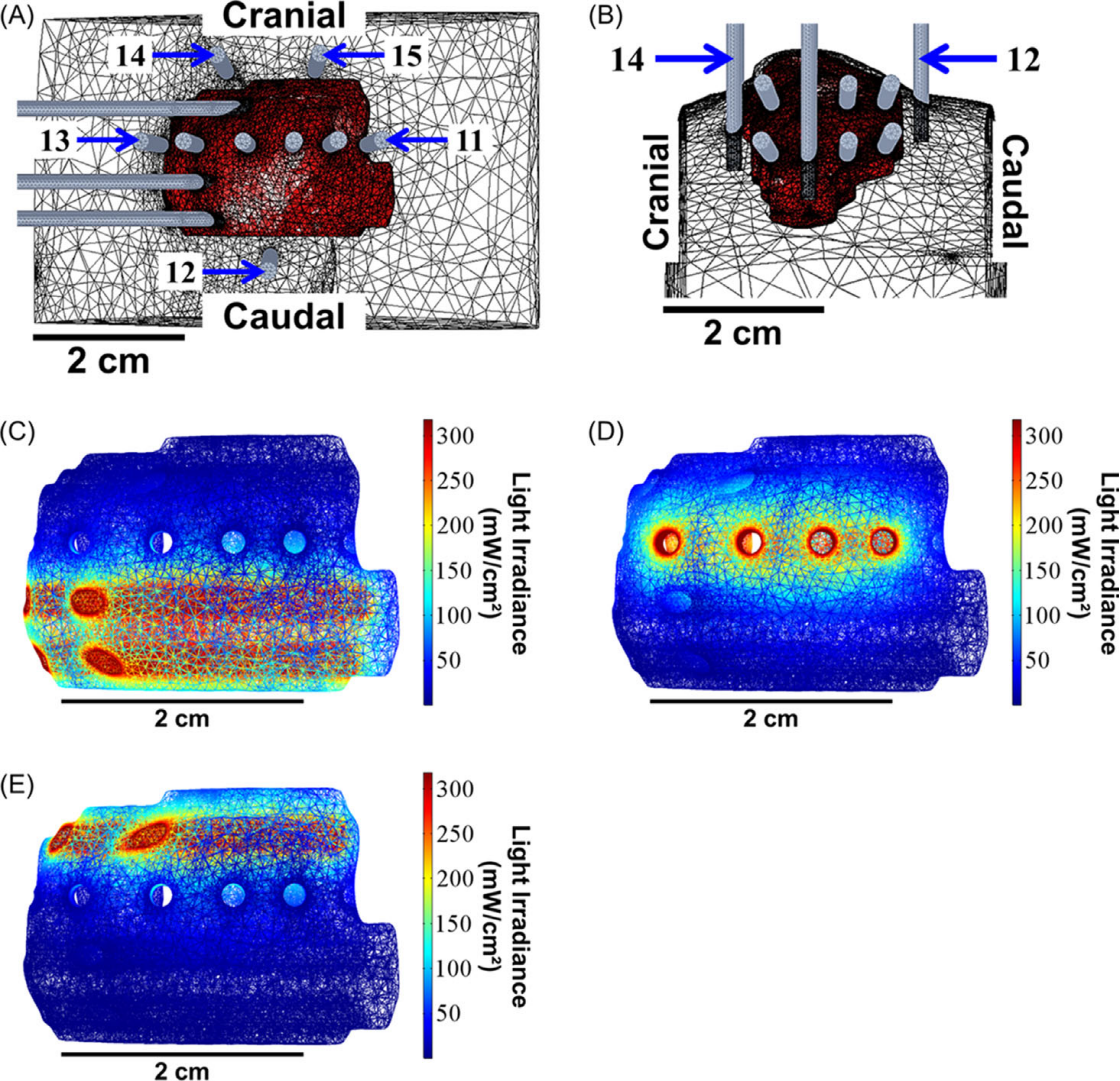
Treatment plan for RB2018‐30. (A,B) show the 3D representation of the treatment plan. The tumor is represented in red. The plan was to use catheters #1–10 for light source diffusing fibers and catheters #11–15 for IP85 detection fibers. (C‐E) show the resulting FEM‐simulated distribution of irradiance. For this VX2 tumor, the entire treatment was broken up into three sessions of consecutive light illumination. (C‐E) show the light distribution for each session.

**Figure 4:**
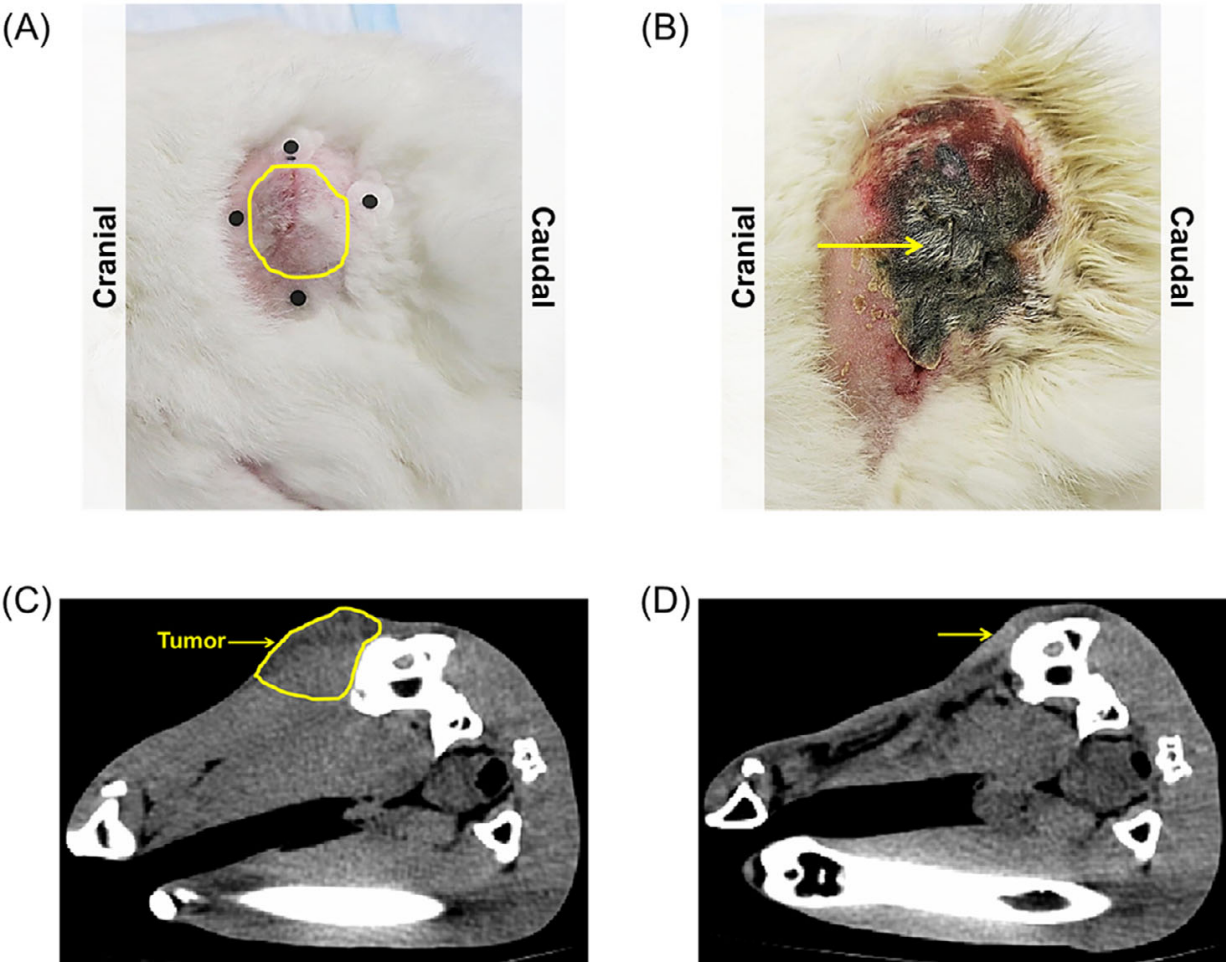
RB2018‐30 tumor response after Photofrin^®^‐mediated I‐PDT. (A) Image of the tumor 1 day prior to treatment. (B) Image of tumor 6 days post‐treatment. (C) and (D) are, respectively, CT scans of the tumor 1 day prior to and 13 weeks post‐treatment. The yellow arrow indicates where the tumor was located in the CT scans.

The next step of this study was to translate our results from the mouse study into a larger animal model, VX2 carcinoma in NZW rabbits. In our mouse studies, we showed that a threshold light irradiance and fluence of, respectively, ≥8.4 mW cm^−2^ and ≥45 J cm^−2^ to 100% of the tumor volume is required for an effective and safe Photofrin^®^‐mediated I‐PDT. To translate this effective light regimen in the treatment of the larger, locally advanced VX2 tumors, we applied our FEM to generate individualized treatment planning for each rabbit. The FEM simulations suggested that between 4 and 12 source diffusing fibers delivering between 200 and 250 mW cm^−1^ per fiber were required to deliver the threshold irradiance of ≥8.4 mW cm^−2^ in 100% of the tumor volume. Fig. 3 shows a representative example of the FEM‐based treatment plan. Figure 3A and B shows the treatment plan developed for a rabbit RB2018‐30 that was treated with 5 mg kg^−1^ Photofrin^®^‐mediated I‐PDT, which is equivalent to a 2 mg kg^−1^ dose in humans. The tumor treatment volume for this rabbit was 5600 mm^3^. The plan was to insert 10 catheters that would be used during treatment for light‐diffusing fibers and 5 catheters that would be used during treatment for IP85 detection fibers to monitor the irradiance and fluence delivered to the margins of the tumor. The light treatment for this animal was conducted in three sessions of consecutive light illumination. Figure 3C–E shows the resulting FEM‐simulated distribution of irradiance for each treatment session. Based on our FEM simulation, a threshold irradiance of 19.7 mW cm^−2^ should have been delivered to 100% of the tumor volume. The catheters were placed in the VX2 tumor based on the FEM treatment planning using the CT fiducial markers as guides. It was important to measure the irradiance and fluence throughout the tumor and margins during treatment using our light dosimetry system due to differences in the actual catheter location and the FEM planned location as well as differences in the actual tissue optical properties and those used for our FEM simulations. From our dosimetry measurements, it was observed that during the first two treatment sessions, both catheter 11 and 12 did not obtain the effective threshold irradiance and fluence that was suggested by our mouse studies (≥8.4 mW cm^−2^ and ≥45 J cm^−2^). Therefore, in the final treatment session for RB2018‐30, an additional two light source diffuser fibers were added to catheters 11 and 12. Figure 4 depicts the response to 5 mg kg^−1^ Photofrin^®^‐mediated I‐PDT for RB2018‐30. Figure 4A is an image of the tumor 1 day prior to I‐PDT. Within a week after I‐PDT, a scab began to form at the treatment site (see Fig. 4B). This scab typically fell off within 4–6 weeks after I‐PDT and the skin began to heal. Weekly noncontrast‐enhanced CT scans were taken 2–3 weeks after I‐PDT to monitor the treatment site for tumor recurrence and the lungs for metastasis. According to our IACUC protocol, to avoid adverse events, rabbits with lung metastasis were euthanized 1–2 days post‐treatment. If lung metastasis or other complications such as infection occurred following treatment, the tumor treatment area was examined for the presence of any remaining viable tumor tissue. If there was no evidence of disease or morbidity, the animal was followed for at least 12 weeks post‐treatment. The rabbit in Figs 3, 4 survived 13 weeks post‐treatment with no sign of tumor recurrence or lung metastasis indicating that this animal had complete response and was declared a cure. A summary of results from all of the rabbits treated in this study is given in Table 3. Of the six rabbits with VX2 in the neck treated with 5 mg kg^−1^ Photofrin^®^‐mediated I‐PDT, 2 rabbits were cures, 3 had local control, and 1 had local response. Of the 8 rabbits with VX2 in the thigh treated with 5 mg kg^−1^ Photofrin^®^‐mediated I‐PDT, 4 rabbits were cures and the other 4 had local control. For the one rabbit that exhibited only local response with regional metastasis to the salivary glands (RB2017‐23), the FEM‐simulated threshold irradiance was 6.9 mW cm^−2^. We assume that local tumor control was not obtained in this animal due to the low threshold light irradiance.

**Table 3:** Summary of results from I‐PDT of locally advanced VX2 Carcinoma.

Rabbit #	Tumor Location	Tumor Volume (mm^3^)	Photofrin^®^ Dose (mg kg^−1^)	FEM‐Simulated Threshold Irradiance (mW cm^−2^)	Response
RB2017‐23	Neck	10 900 (10.9 cm^3^)	5	6.9	Local response with regional metastasis to salivary glands and evidence of lung metastasis.
RB2017‐24	Neck	5200 (5.2 cm^3^)	5	30.5	Cure—No evidence of tumor at 13 weeks post‐therapy.
RB2017‐25	Neck	6200 (6.2 cm^3^)	5	25.8	Cure—No evidence of tumor at 13 weeks post‐therapy.
RB2017‐30	Neck	5700 (5.7 cm^3^)	5	16.5	Local control. Lung metastasis.
RB2017‐31	Neck	14 800 (14.8 cm^3^)	5	22.9	Local control. Lung metastasis prior to treatment.
RB2018‐03	Neck	5500 (5.5 cm^3^)	5	23.4	Local control. Lung metastasis.
RB2018‐02	Thigh	5500 (5.5 cm^3^)	5	30.5	Local control. Infection in the leg. No lung metastasis.
RB2018‐17	Thigh	3200 (3.2 cm^3^)	5	24.8	Cure—No evidence of tumor at 13 weeks post‐therapy.
RB2018‐18	Thigh	5800 (5.8 cm^3^)	5	15.3	Local control. Lung metastasis prior to treatment.
RB2018‐22	Thigh	7200 (7.2 cm^3^)	5	17.6	Local control. Lung metastasis prior to treatment.
RB2018‐28	Thigh	3500 (3.5 cm^3^)	5	31.3	Local control. Nerve damage. Lung metastasis.
RB2018‐30	Thigh	5600 (5.6 cm^3^)	5	19.7	Cure—No evidence of tumor at 13 weeks post‐therapy.
RB2018‐31	Thigh	2700 (2.7 cm^3^)	5	21.4	Cure—No evidence of tumor at 13 weeks post‐therapy.
RB2018‐32	Thigh	6400 (6.4 cm^3^)	5	18.8	Cure—No evidence of tumor at 13 weeks post‐therapy.
RB2017‐27	Neck	6400 (6.4 cm^3^)	0	12.8	Died immediately post‐treatment.
RB2017‐28	Neck	3200 (3.2 cm^3^)	0	19.7	Progressive disease
RB2017‐29	Neck	6800 (6.8 cm^3^)	0	19.0	Died immediately post‐treatment.
RB2018‐14	Thigh	5300 (5.2 cm^3^)	0	28.9	Progressive disease
RB2018‐19	Thigh	11 300 (11.3 cm^3^)	0	23.0	Progressive disease

The same treatment planning was conducted for the rabbits with VX2 treated with light only (*n* = 5). Of these 5 animals, in 3 we observed progressive disease and the other 2 died immediately post‐treatment due to aspiration pneumonia which is a possible complication in NZW rabbits while under anesthesia.

## Discussion

In a recent preclinical light dose‐finding study for Photofrin^®^‐mediated I‐PDT, we demonstrated the importance of irradiance in obtaining tumor control and cures in the treatment of locally advanced cancers with Photofrin^®^ 3. In the study reported here, we further demonstrated the importance of irradiance during Photofrin^®^‐mediated I‐PDT by treating mice with FEM‐simulated subtherapeutic and therapeutic irradiances. In all cases, the same minimum fluence of 45 J cm^−2^ was administered. In the first cohort of mice with SCCVII tumors, we intentionally lowered the threshold irradiance to 5.4 mW cm^−2^ while still maintaining the input intensity per fiber at 100 mW cm^−1^ (the input light intensity that resulted in the 70% cure rate in our previous studies). This was done by delivering the light from each fiber consecutively (one fiber at a time) rather than simultaneously as we had previously done. When 100 mW cm^−1^ was delivered consecutively from two 2 cm diffuser fibers thereby delivering a threshold of 5.4 mW cm^−2^ to 100% of the tumor volume, no cures were observed with and without Photofrin^®^. We attributed the zero cures to the low threshold light irradiance in comparison to the recommended curative threshold (i.e. 5.4 *vs* 8.4 mW cm^−2^). We then increased the input light intensity to 160 and 200 mW cm^−1^ per fiber delivered consecutively, which based on our FEM simulations would result in threshold light irradiances of, respectively, 8.6 and 10.8 mW cm^−2^ (the same minimum total fluence of 45 J cm^−2^ was delivered). At these light settings, between 20% and 90% cures were observed. As with our previous study, we did observe cures in mice treated with light only indicative of thermal ablation; however based on this study, there is still a higher probability of obtaining local tumor control when Photofrin^®^ is administered (60% *vs* 15%). The impact of thermal ablation during I‐PDT on tumor response has not been studied in the work reported here. Future works aim at further developing a dosing scheme for the irradiance and fluence that would optimize the photoreaction and thermal ablation effects during I‐PDT in the effort of further improving the control of locally advanced cancers. Ultimately, we aim to develop a predictive model for I‐PDT. The statistical analysis provided here supports the combination of the irradiance and initial tumor volume as predictors for tumor response to Photofrin^®^ mediated I‐PDT.

In the second part of this study, we applied our FEM to translate our findings from the previous and current mouse studies to treat locally advanced VX2 tumors in NZW rabbits with Photofrin^®^‐mediated I‐PDT. The goal was to treat these tumors with a threshold irradiance and fluence of 8.4 mW cm^−2^ and 45 J cm^−2^. Our FEM simulations suggested that between 4 and 12 light diffuser fibers each delivering between 200 and 250 mW cm^−1^ were required to deliver our threshold light irradiance and fluence and achieve tumor margin control. On average, the FEM‐simulated threshold irradiance was 21.5 ± 6.2 mW cm^−2^ and the maximum irradiance delivered was between 318.3 and 397.9 mW cm^−2^.

Using our light dosimetry system, we monitored the irradiance and fluence during I‐PDT near and at the tumor margins, to minimize under‐ or overtreatment. In cases where the measured irradiance and fluence were too low, we adjusted the treatment by moving the laser fibers to catheters where low irradiance was measured. This method resulted in complete ablation of the VX2 tumor. By monitoring the change in irradiance, we can also account for differences between the actual catheter location and the planned catheter location and between the actual tissue optical properties and those used in our FEM simulations, as we rely on light dosimetry to deliver the prescribed threshold light irradiance and fluence. We were also able to minimize the number of catheters needed for these dosimetry measurements by using consecutive light illumination and inserting the IP85 detection fibers in catheters that were designated for light diffuser fibers during a different treatment session.

Of the 14 rabbits treated with 5 mg kg^−1^ Photofrin^®^, 6 were cured, 7 achieved local control, and 1 exhibited local response, but no control. In the one rabbit that only had local response and developed metastasis to the salivary gland, the FEM‐simulated threshold light irradiance was 6.9 mW cm^−2^ which could explain why this rabbit did not have local tumor control. In contrast to the treatment of locally advanced SCCVII tumors in C3H mice, the large VX2 tumors did not have any tumor growth delay when treated with light only. We hypothesize that this difference was due to either a difference in vasculature and/or animal size. Both the vasculature and the larger body of the rabbit (2400–3000 g *vs* 25 g) could act as a heat sink and dissipate the light‐induced temperature from the tumor volume 20, 21. Ongoing work aims at defining the impact of thermal ablation during I‐PDT in locally advanced tumors in large animals.

## Conclusion

The study reported here demonstrated that using treatment planning to guide consecutive light illumination with light dosimetry enables careful control of Photofrin^®^ mediated I‐PDT of locally advanced tumors. Our results demonstrated that for the same threshold intratumoral fluence, the intratumoral threshold irradiance is a key parameter in obtaining high cure rate that is in agreement with our previously reported study (Shafirstein et al. 2018). Our results and analysis suggest that the irradiance and initial tumor volume can be used to accurately predict tumor response (i.e. cure or no cure) to Photofrin^®^ mediated I‐PDT in a translational mouse model. The contribution of the light‐induced tissue heating appears to be minimal in the treatment of large tumors (i.e.VX2 in the NZW rabbits).

## Conflict of interest

Gal Shafirstein, David Bellnier and Emily Oakley are coinventors in patent applications owned by Roswell Park Cancer Institute of a light dosimetry system for interstitial and thermal photodynamic therapy. Gal Shafirstein acknowledges research grant support from Concordia Laboratories Inc. Gal Shafirstein acknowledges a service on the advisory board for Concordia International Corp. and Pinnacle Biologics, Inc. All other coauthors declare no potential conflicts of interest.
